# Hitting the target or missing the point? A closer look at post–cardiac arrest guideline adherence

**DOI:** 10.1186/s13613-025-01560-x

**Published:** 2025-09-25

**Authors:** Giulia Merigo, Fabiana Madotto, Aurora Magliocca, Giuseppe Ristagno

**Affiliations:** 1 Department of Pathophysiology and Transplantation, University of Milan, Milan, Italy; 2 Department of Anesthesiology, Intensive Care and Emergency, Fondazione IRCCS Ca’ Granda Ospedale Maggiore Policlinico, Via Francesco Sforza 35, Milan, 20122 Italy

We thank Dr. Mannu [1] for his interest in our recent study on adherence to post-cardiac arrest care guidelines and its association with patients’ outcomes [2]. His comments highlight several important considerations that deserve further discussion.

Dr. Mannu correctly identified a key methodological challenge in evaluating guidelines adherence, i.e. our metrics were based on achieved physiological targets rather than direct documentation of clinical intent. We acknowledge that this approach may have not fully captured the complexity of clinical decision-making. As we noted in our manuscript, failure to achieve a physiological target does not necessarily imply a lack of adherence to guideline recommendations. This is particularly relevant in cases of refractory shock, where appropriate treatments might have been administered but proved insufficient due to patient’s clinical severity. In such scenarios, adherence metrics might underestimate clinician effort and the severity of the underlying illness. To further explore this issue, we are currently conducting dedicated analyses to identify factors associated with non-adherence to guideline targets, accounting for both patient-related variables (e.g., clinical instability, disease severity) and treatment-related decisions/interventions. While the full results will be presented in a subsequent manuscript, we anticipate some preliminary findings here related to hemodynamic adherence. Specifically, among patients classified as adherent to mean arterial pressure (MAP) targets, approximately half required no more than one vasopressor at any given timepoint (24, 48, 72, and 96 h). Conversely, about half of those classified as non-adherent consistently required two or more vasopressors (Fig. 1). This pattern suggests that even within the non-adherent group, substantial clinical efforts were undertaken by clinicians to achieve the guideline targets; efforts which were likely hindered by greater patient’s hemodynamic instability. These findings underscore the need for more accurate interpretation of adherence in critically ill populations. As Dr. Mannu appropriately noted, this applies not only to hemodynamic management but also to other domains such as ventilation, glycaemic control, and additional guideline-based targets. These broader aspects will be addressed in forthcoming analyses and manuscripts.

**Fig. 1:**
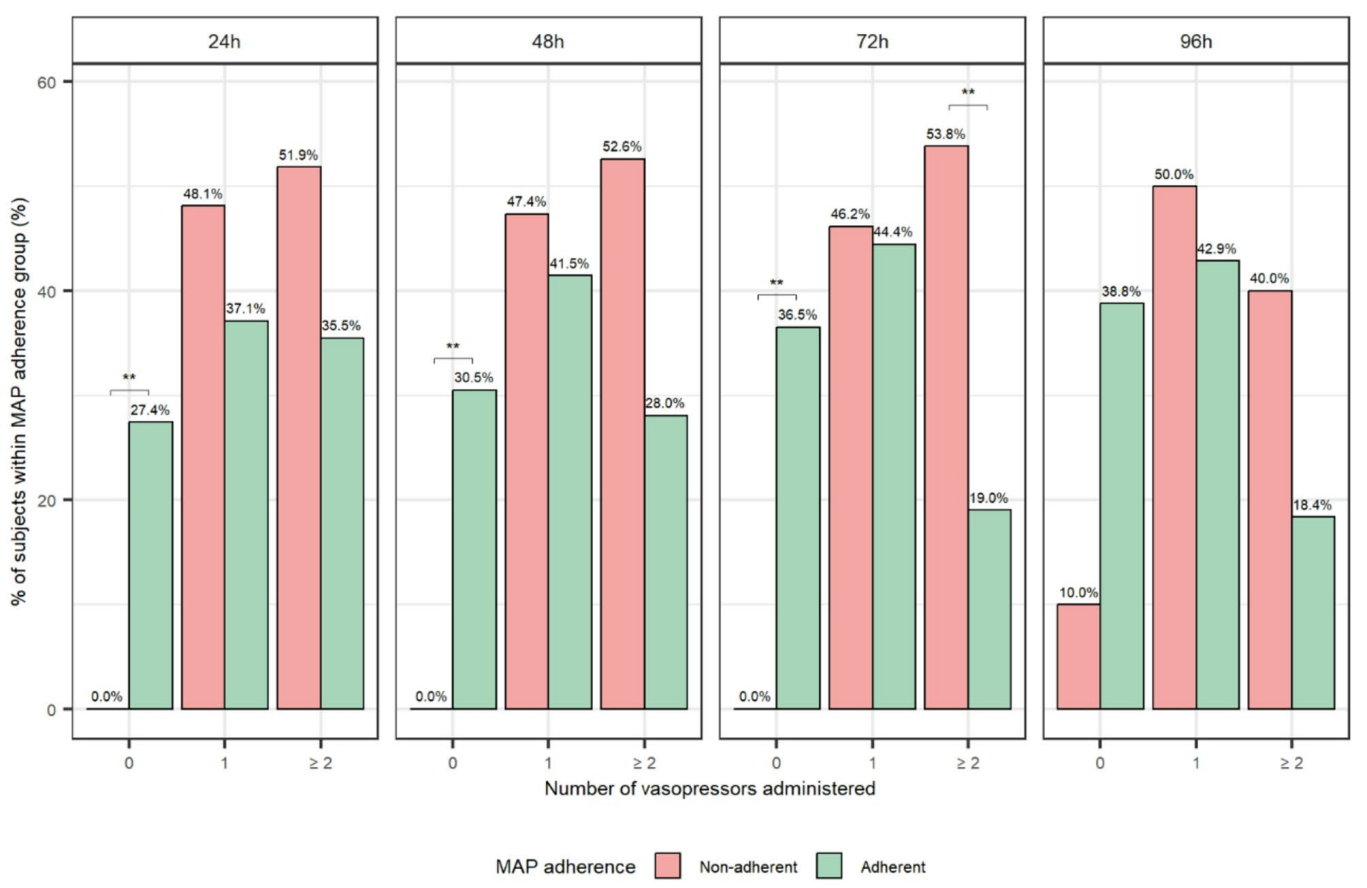
Distribution of vasopressor administration by mean arterial pressure (MAP) adherence status over time in intensive care unit (ICU). Bar chart showing the percentage of patients within each MAP adherence group (adherent vs. non‑adherent), stratified by the number of vasopressors administered (0, 1, or ≥ 2) and by post‑ICU admission timepoints (24, 48, 72, 96 h). Adherent patients are shown in green and non‑adherent patients in red. * indicates statistically significant differences between MAP adherence groups for each vasopressor category, based on pairwise post-hoc comparisons adjusted using the Bonferroni method (*p* < 0.05)

Prompted by Dr. Mannu comment regarding improved survival rates in recent years, we reanalysed temporal trends in outcomes. As illustrated in Fig. 2, both survival and neurological recovery have significantly improved over time, likely reflecting not only increased adherence to guidelines but also general advancements in critical care. Interestingly, outcome improvements appeared to follow a specific temporal pattern, with notable gains occurring shortly after the introduction of updated guidelines, peaking just before the release of subsequent recommendations [3, 4]. An exception was observed in 2020, likely attributable to the impact of the COVID-19 pandemic [5]. In our original analysis, we accounted for these temporal changes by incorporating the guideline cohorts (2011–2015, 2016–2020, and 2021–2024) as a predictor variable for clinical outcomes. Indeed, the cohort effect was identified as an independent predictor of favourable neurological outcomes at intensive care unit (ICU) discharge. Of note, we decided to use the cohort as an adjustment variable rather than calendar year, either as a continuous or categorical variable. This strategy allowed us to control for evolving practices without assuming a strictly linear relationship between time and outcomes (as would have been required if using calendar year as a continuous variable), and it minimized the risk of overfitting, while preserving model stability, associated with a fully categorical time variable, especially given the number of years involved. Notably, the cohort effect emerged as an independent predictor of favourable neurological outcome at ICU discharge.

Finally, regarding implementation efforts, we confirm that throughout the study period, our centre periodically revised and updated internal protocols for post-resuscitation care to align with evolving guidelines and emerging evidence. Education initiatives, including structured training programs for physicians and nurses, were part of our local continuous process of care improvement. A designated physician served as the lead for implementation, ensuring timely updates and dissemination of best practices. These systematic strategies likely contributed to the observed improvements in adherence and outcomes over time, as reflected in Fig. 2.

In conclusion, we appreciated Dr. Mannu’s insightful contributions and fully concur with his call for further research, particularly multicentre trials, to more precisely delineate the impact of post-cardiac arrest guideline adherence on long-term outcomes in resuscitated patients.

**Fig. 2:**
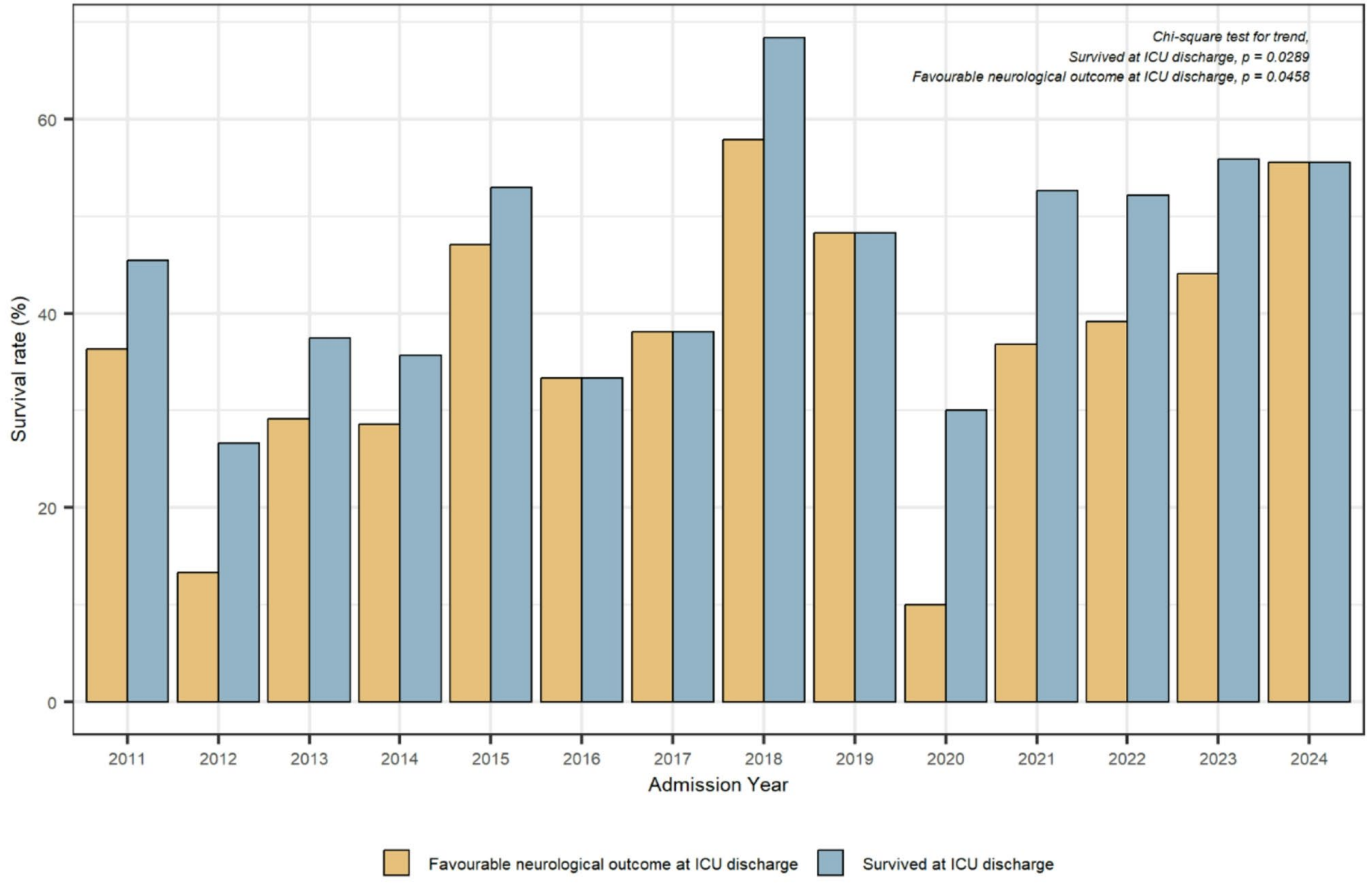
Temporal trends in survival and neurological recovery at intensive care unit (ICU) discharge. Bar plot showing annual trends in survival rates at ICU discharge (light blue) and favourable neurological outcomes (CPC 1–2; light yellow) among patients admitted from 2011 to 2024. Rates are expressed as percentages of total cases per year. Both outcomes show statistically significant trends over time (Chi-square tests for trend: ICU survival, *p* = 0.0289; neurological outcome, *p* = 0.0458)

## Data Availability

Not applicable.

## Abbreviations

MAP: Mean arterial pressure
ICU: Intensive care unit
